# Infant Feeding and Ethnic Differences in Body Mass Index during Childhood: A Prospective Study

**DOI:** 10.3390/nu13072291

**Published:** 2021-07-01

**Authors:** Outi Sirkka, Tanja Vrijkotte, Lieke van Houtum, Marieke Abrahamse-Berkeveld, Jutka Halberstadt, Margreet R. Olthof, Jacob C. Seidell

**Affiliations:** 1 Department of Health Sciences, Faculty of Science, Amsterdam Public Health Research Institute, Vrije Universiteit Amsterdam, 1081 HV Amsterdam, The Netherlands; j.halberstadt@vu.nl (J.H.); margreet.olthof@vu.nl (M.R.O.); j.c.seidell@vu.nl (J.C.S.); 2 Danone Nutricia Research, 3584 CT Utrecht, The Netherlands; Marieke.Abrahamse@danone.com; 3 Department of Public and Occupational Health, Amsterdam Public Health Research Institute, Amsterdam UMC, University of Amsterdam, 1105 AZ Amsterdam, The Netherlands; t.vrijkotte@amsterdamumc.nl; 4 Sarphati Amsterdam, Municipal Health Service Amsterdam, 1018 WT Amsterdam, The Netherlands; lvhoutum@ggd.amsterdam.nl

**Keywords:** infant feeding, BMI, ethnicity, breastfeeding, complementary feeding

## Abstract

This study investigated ethnic differences in childhood body mass index (BMI) in children from Dutch and Turkish descent and the role of infant feeding factors (breastfeeding duration, milk feeding frequency, as well as the timing, frequency and variety of complementary feeding (CF)). We used data from 244 children (116 Dutch and 128 Turkish) participating in a prospective study in the Netherlands. BMI was measured at 2, 3 and 5 years and standard deviation scores (sds) were derived using WHO references. Using linear mixed regression analyses, we examined ethnic differences in BMI-sds between 2 and 5 years, and the role of infant feeding in separate models including milk or CF factors, or both (full model). Relative to Dutch children, Turkish children had higher BMI-sds at age 3 (mean difference: 0.26; 95%CI: 0.04, 0.48) and 5 (0.63; 0.39, 0.88), but not at 2 years (0.08; −0.16, 0.31). Ethnic differences in BMI-sds were somewhat attenuated by CF factors at age 3 (0.16; −0.07, 0.40) and 5 years (0.50; 0.24, 0.77), whereas milk feeding had a minor impact. Of all factors, only CF variety was associated with BMI-sds in the full model. CF factors, particularly CF variety, explain a small fraction of the BMI-sds differences between Dutch and Turkish children. The role of CF variety on childhood BMI requires further investigation.

## 1. Introduction

Childhood overweight (and obesity) remains an important and growing global public health issue [1]. In the Netherlands, overweight rates have slightly declined during recent years among children from Dutch descent, while remaining high among children from Turkish descent [2,3,4]. Infants of Turkish descent have been reported to have a higher body mass index (BMI) already during the first year of life [5], and by 2 years of age, one out of five children of Turkish descent has developed overweight [3]. Infant feeding has been suggested as one of the most important modifiable risk factors for long-term growth outcomes [6,7,8]. Hence, a better understanding of the role of infant feeding in explaining ethnic differences in childhood BMI is pivotal.

Previous studies on infant feeding and weight-related outcomes have mainly focused on (exclusive) breastfeeding (BF) duration, and the timing of introduction of complementary feeding (CF), with somewhat conflicting results [9,10,11]. Although some studies have reported that a longer BF duration and a later introduction of CF are associated with a lower BMI during childhood [7,12] others reported no associations [13,14]. To our knowledge, only limited evidence exists on other infant feeding factors, such as feeding frequency or food variety [15], i.e., the number of food groups consumed. Although a high (milk) feeding frequency has been associated with infant weight gain [16], no studies have assessed the long-term impact of either milk- or CF frequency during infancy on childhood BMI development. Moreover, although several aspects of milk- and CF feeding may play a role in the development of overweight, previous studies have not investigated their role independently of other feeding factors.

Large ethnic differences exist in infant feeding [17,18]. In the Netherlands, infants from Turkish descent generally receive a longer duration of BF and are introduced to CF later than Dutch infants [18]. Although previous studies in the Netherlands including Dutch infants reported associations of BF duration and the timing of CF with infant weight gain [18], or higher BMI during childhood [19], these studies found no associations among Turkish infants. Previously, in the cohort studied in the current study, Turkish infants were found to receive more frequent feedings and a higher variety of complementary foods than Dutch infants at 6 months of age [20]. It is of interest to investigate the long-term associations of infant feeding practices with childhood growth outcomes in these populations.

The aim of the current study was to investigate the contribution of infant feeding factors to the ethnic differences in BMI between 2 and 5 years in children of Dutch and Turkish descent. We examined the contribution of following infant feeding factors: BF duration, milk feeding frequency as well as the timing, frequency and variety of CF. Our hypothesis was that infants of Turkish descent would show increase in BMI development compared to infants of Dutch descent, which would be largely explained by infant feeding factors related to CF.

## 2. Materials and Methods

### 2.1. Subjects

The inclusion of the study population for the current study has been described previously in detail [20]. In short, the study population includes Turkish and Dutch mothers and their infants, born between August 2009 and March 2010, in the city of Amsterdam. Infants were considered to be of Dutch descent when both parents and the mothers’ mother were born in the Netherlands. If at least one parent and the mothers’ mother were born in Turkey, infants were considered of Turkish descent [20,21]. Inclusion criteria for participation were: term gestational age (≥37 weeks), appropriate weight for gestational age (≥10th percentile of the Dutch national reference curves), singleton pregnancy, no maternal complications during pregnancy (e.g., no diabetes or hypertension) and no medical problems diagnosed during pregnancy or after birth (e.g., congenital malformations). Approximately 2 weeks after birth, when data on ethnicity, pregnancy, birth and neonatal health were recorded in the Youth Health Care (YHC) registration, mothers who met the inclusion criteria were contacted by phone and provided with verbal and written study information. Once participation was agreed, a home visit was made 4 weeks after birth, during which written informed consent was obtained. In total, 368 mothers were asked to participate, of whom 300 (150 Dutch and 150 Turkish) agreed (81.5%) to participate in the follow-up, which included three home visits (1, 4 and 6 months after birth). Of these mothers, 286 completed the data collection during the home visits. The sociodemographic characteristics of the fourteen mothers that dropped out were similar to the characteristics of the participating mothers. Children with complete data on the feeding factors at 6 months and ≥1 measure of BMI-sds during 2–5 years of age were included in the analysis. The final study sample consisted of 244 children, 116 from Dutch and 128 from Turkish descent (Figure S1). The study protocol was approved by the Medical Ethical Committee of the Academic Medical Centre in Amsterdam, The Netherlands.

### 2.2. Data Collection

Data on maternal and infant characteristics, as well as infant feeding, were collected during home visits when infants were 1, 4 and 6 months old by means of a standardized questionnaire and a 24-h recall. Home visits were made by a team of ten medical students, who were carefully selected and trained to secure the quality of the data. Students were able to speak Turkish if requested by the mother.

#### 2.2.1. Infant Feeding

During the home visits, mothers were asked to provide information on the feeding of their infant by filling out a standardized questionnaire. A 24-h food recall was also performed to obtain data on feeding from the day preceding the visit. The questionnaire assessed the duration of BF, introduction of infant formula, timing of CF and the types of complementary foods introduced. For the duration of BF, mothers were asked to provide information about whether the child had received either BF or formula feeding during the period preceding the home visit and the duration in months. The duration of full BF (BF without additional formula feeding) was then defined based on the duration of full BF and the age of the child when formula feeding was introduced. For the present study, the duration of full BF was dichotomized as <6 months and ≥6 months. We chose to investigate full BF (defined as no formula, but other foods/drinks are allowed) instead of exclusive BF (no formula or other foods or drinks) because we were interested to study the role of full BF duration and the timing of CF as separate factors.

Regarding the timing of CF, mothers were asked if they had introduced any complementary foods or drinks to their infant during the period preceding the home visit. Complementary foods were defined as any other liquids or foods other than breastmilk, infant formula or water. For the present study, the timing of CF was dichotomized as <6 months or ≥6 months, according to the WHO recommendations [22].

Data on milk and CF frequency were obtained via the 24-h food recall at 6 months of age. For both feeding factors, the frequency was defined as the number of times these liquids/foods were provided to the infant during the 24-h recall period. Milk feeding frequency included all milk feedings (either infant formula or BF) provided during the 24 h. This also included expressed breastmilk fed from the bottle. Formula feedings with added rice flour to the bottle were also considered as milk feedings. For the CF frequency, all liquids or foods other than breast milk, infant formula (with or without added rice flour) or water, were included.

CF variety was assessed at 6 months of age by means of the standardized questionnaire by asking the mothers which types of complementary foods they had introduced to their infants. All mothers had indicated that they had started CF by the time of the 6-month home visit. Additionally, information on the types of complementary foods mentioned in the 24 h-recalls was used to complement the data from the questionnaire. Data on the types of complementary foods were grouped into 5 food groups: (1) fruits and vegetables, (2) dairy products, (3) bread, cereals and rusks (4) cookies and (5) savory foods. The food group “Fruits and vegetables” included all food items consisting of vegetables or/and fruits only, including commercial jars. “Dairy products” included cow’s milk, cheese and yogurt. “Bread, cereals and rusks” included breads, infant cereal meals and porridge as well as rice crackers, rusks and breadsticks. “Cookies” included all types of cookies, including baby cookies. “Savory foods” included all savory (mixed) meals and snacks which were not included in the other food groups, such as meat and pizza. We created a CF variety score based on the number of food groups consumed by the infant, by assigning each food group with one point. The CF variety score was then calculated as the total number of food groups. For the purpose of the analysis, the CF variety score was categorized then as: 1, 2 or ≥3. In case no information on the types of foods was available either from the questionnaire or the 24 h-recall, these data were considered missing.

#### 2.2.2. BMI during Childhood

Information on the child’s weight and height during childhood was obtained from the YHC centers, where height and weight were measured during standard health examinations at 2, 3 and 5 years of age. BMI was calculated as weight in kilograms divided by the square of height in meters (kg/m^2^). On average, 2.6 BMI measurements were available for each child. BMI values were then transformed into standard deviation scores (BMI-sds) according to the WHO age- and sex-specific growth references for 2, 3 and 5 years of age [23,24]. For the calculation of BMI-sds, Growth Analyser Software version 4.0 (Growth Analyser BV), was used. Overweight was defined as >+1 SD above the median of the BMI references at 2 and 3 years, and >2+ SD at 5 years, respectively.

#### 2.2.3. Covariates

We included the following covariates: BMI, education, age, parity and smoking of the mother, as well as gestational age and birth weight of the infant. For maternal BMI, height and weight data were measured by physicians at the YHC centers during the regular 6-month examination, which all children receive. Maternal BMI was dichotomized as either normal weight: BMI < 25 kg/m^2^, or overweight (including obesity): BMI ≥ 25 kg/m^2^. Maternal educational level was recorded during the at 4-month home visit, and categorized based on the years of education: low (<6 years); medium (6–10) or high (≥10 years). Information on maternal age (years) was obtained from the YHC registration. Parity (primiparity/multiparity) and smoking during pregnancy (yes/no) were recorded during the 1-month home visit. Data on infant’s gestational age (in days), birth weight (in grams) and sex were obtained from the YHC registration. For birth weight, sds was calculated according to the WHO reference values [25]. For this, the Growth Analyser Software (version 4.0) ( Rotterdam, The Netherlands) was used.

### 2.3. Statistical Analysis

We used linear mixed regression (LMR) analysis, including both fixed and random effects (random intercept for each subject), to examine ethnic differences in BMI-sds between 2 and 5 years, and the explanatory role of infant feeding factors. This analysis takes into account the repeated BMI-sds measurements per subject and overcomes challenges inherent to longitudinal studies: bias due to missing data and the requirement for all individuals to be measured at the same ages.

First, to answer our first research question, in model 1 (crude model) potential ethnic differences in BMI-sds between 2 and 5 years were estimated by including age and the ethnicity variables as the main effects. We also assessed whether the potential ethnic differences in BMI-sds remained similar over time during the period of 2–5 years by including an interaction term between ethnicity and age in the crude model. In case of a significant interaction, the interaction term was included in the subsequent models and marginal means and 95% CIs were generated from the LMR models to demonstrate differences in BMI-sds at each age separately.

Second, to investigate the role of milk and CF factors in explaining the association between ethnicity and BMI-sds, separate models (models 2–4) were applied. Model 2 included model 1 plus the milk feeding factors full BF duration and milk feeding frequency. Model 3 included model 1 plus the CF factors timing of CF, CF variety and CF frequency. Model 4 included all feeding factors (models 1 + 2 + 3) to investigate the milk and CF factors simultaneously and their independent association with BMI-sds.

We additionally examined potential ethnic differences in the associations between feeding factors and BMI-sds to understand whether the impact of feeding factors on BMI-sds are similar for both ethnic groups. This was done by including interaction terms between ethnicity and each feeding factor in models 2 and 3. Additionally, we considered whether associations between each feeding factor and BMI-sds varied by age. This was done by including interaction terms between each feeding factor and age in models 2 and 3. In the case of significant interactions (*p* < 0.05), the terms were included in the subsequent models.

An additional adjusted model (model 5, including model 4 + covariates) was fitted including the subset of population with complete data on the pre-defined maternal and infant covariates: maternal age, parity and smoking, as well as infant’s gestational age and birth weight (n = 229), Table S1. Maternal BMI was not included in model 5 analyses due to a substantial amount of missing data for maternal BMI (>30%). As a sensitivity analysis, model 6 assessed potential confounding by maternal BMI. This was done by including maternal BMI in the model in addition to the model 5 covariates (Table S2). Model 6 included the subset of population with complete data on the covariates and maternal BMI (n = 156). Maternal educational level was not included in models 5 and 6 due to a high correlation with ethnicity [20].

## 3. Results

Apparent differences between maternal and infant characteristics, as well as BMI and the prevalence of overweight at age 5, were observed between the two ethnic groups (Table 1).

### Ethnic Differences in BMI between 2 and 5 Years and the Role of Infant Feeding Factors

There was a significant interaction (*p* < 0.001) between ethnicity and age, suggesting that ethnic differences in BMI-sds are age-specific. In addition, the interaction term for food variety and age was significant (*p* = 0.007), suggesting that the association between food variety and BMI-sds differs depending on the age. Therefore, these interaction terms were included in the subsequent analyses. The results are shown as marginal means and 95% CI’s, which were generated from the LMR to describe the associations at each age.

Table 2 shows the mean differences in the estimated BMI-sds at 2 years by ethnicity and feeding factors, derived from the LMR. Ethnic differences in BMI-sds were not present at 2 years (Table 2, crude model). The addition of milk and CF factors in models 2 and 3 did not change the magnitude of the mean differences in BMI-sds between the ethnic groups. When examining the independent associations of all the feeding factors with BMI-sds in the full model (model 4), only CF variety was significantly associated with BMI-sds at age 2; children with a CF variety of ≥3 had a 0.39 units higher BMI-sds than those with a CF variety of 1.

At 3 years, Turkish children had statistically significant 0.26 units higher BMI-sds compared to Dutch children (Table 3, crude model). Milk feeding factors slightly reduced the ethnic differences in model 2. In model 3, after the adjustment for CF factors, ethnic differences attenuated (0.10 sds units), and became non-significant. When all the feeding factors were mutually adjusted for in model 4, CF variety of ≥3 (vs. CF variety of 1) was associated with BMI-sds (mean difference: 0.44; 95% CI: 0.10, 0.78) independent of other feeding factors and ethnicity.

Ethnic differences in BMI-sds were even more apparent at 5 years of age; Turkish children had a statistically significant 0.63 units higher BMI-sds compared to Dutch children (Table 4, model 1). Although CF factors attenuated the ethnic differences (0.13 sds units), ethnic differences remained significant also in models 3 and 4. In model 4, only CF variety (≥3 vs. 1) was significantly associated with BMI-sds (mean difference: 0.55; 95% CI: 0.17, 0.92), independent of other feeding factors and ethnicity.

Results from the sensitivity analyses including adjustment for maternal and infant covariates (model 5, Table S1), and additionally for maternal BMI (model 6, Table S2), showed further reduction in the ethnic differences in BMI-sds compared to model 4 results at each age (Table 2, Table 3 and Table 4). Furthermore, in models 5 and 6, the adjustment for the covariates did not change the associations of the feeding factors with BMI-sds, except for the slight attenuation of the estimates for full BF and CF variety (Tables S1 and S2).

## 4. Discussion

In this longitudinal study, we found that relative to Dutch children, Turkish children had a higher BMI-sds at age 3 and 5 years, but not at 2 years. A small fraction of the ethnic differences in BMI-sds were explained by CF factors, particularly CF variety, whereas milk feeding factors had a minor impact.

We observed that the ethnic differences in BMI increased with age. Although previous studies in the Netherlands reported higher overweight prevalence at 2 years among children from Turkish descent compared to children from Dutch descent [26,27], we did not observe ethnic differences in BMI at this age. Furthermore, in our study, the observed ethnic differences in BMI at 2 years of age were smaller than those reported by a previous study in the city of Amsterdam [26]. Possible explanations for this include differences in sample size, as well as the inclusion criteria. The current study was relatively small and included singleton infants who were born at term, with an appropriate weight for gestational age and mothers with no complications during pregnancy (e.g., diabetes or hypertension).

To our knowledge, our study is the first in examining the role of several infant feeding factors simultaneously, including CF variety, on ethnic inequalities in childhood BMI at different ages. We found that CF variety explained a small fraction of the ethnic differences in BMI at 3 and 5 years of age. CF variety was also strongly associated with BMI at 2, 3 and 5 years of age, independent of ethnicity and the other feeding factors. There was also some indication that the association between CF variety and BMI increased with age, suggesting that the role of early nutrition may become more apparent at older ages. However, this was less clear in the adjusted models 5 and 6. Our results on CF variety and BMI are in line with a previous study on preschool children in the US, which found that a higher dietary variety was associated with a larger annual increase in BMI-sds during a follow-up of 20 months [15]. Additionally, according to a study of Schack-Nielsen et al. [28], an increasing number of foods introduced between 3 and 6 months of age indicated a higher, although not significant, risk for overweight during adulthood. A possible explanation for the association between CF variety and BMI observed in our study is that children who are exposed to a high CF variety during infancy may have an overall high food intake, which leads to an excessive energy intake and subsequently, to an increased weight gain [28]. Furthermore, the quality of foods is also likely to be an important aspect. In our study, a further exploration of the types of complementary foods provided among the children with a high CF variety (≥3 food groups) showed, that a large majority had been provided with dairy products (81.6%) and savory meals (75.0%). However, among the reference group (children with a CF variety score of 1), only 8.4% and 6.4%, respectively, had been provided with these foods (data not shown). It is thus possible that the observed associations on CF variety and BMI may be explained by the types of complementary foods, and particularly the variety of non-recommended foods [29]. In adults, high variety of foods high in added sugars and solid fats, has been shown to increase the likelihood of excess adiposity [30]. Since a variety of ‘healthy’ foods (e.g., vegetables) may be beneficial, but a large variety of palatable, high-energy dense foods and drinks may be unfavorable, further investigation into ‘healthy’ and ‘unhealthy’ variety is of importance. Currently, the Dutch and the WHO guidelines on CF include only the aspect of the appropriate timing of commencing CF. In light of childhood obesity prevention, information on the variety and the type of CF might thus also be needed.

Conflicting evidence exists regarding the association between BF duration and childhood overweight [17,27,31]. In our study, BF duration did not explain the observed ethnic differences in BMI. These findings are in line with several other multi-ethnic studies, which reported no association between BF duration and growth outcomes among ethnic minority children [17,18,32]. We also did not find evidence that the frequency of milk feeding contributed to the ethnic differences in BMI, despite of the fact that Turkish infants were given more frequent milk feedings than Dutch infants. A possible explanation for this is that in our study, we did not distinguish between the frequency of BF and formula feeding. Although one previous study found an association between milk feeding frequency and increased infant adiposity [16], to our knowledge no previous studies have investigated milk feeding frequency in relation to childhood growth outcomes.

In our study, the timing or frequency of CF did not explain the ethnic differences in BMI, and additionally, no associations with BMI were observed at any age. Our findings on the timing of CF are in line with previous multi-ethnic studies in the UK and in the Netherlands [17,18,33]. Although some studies concluded no associations between the timing of CF with overweight [9,34], other studies have shown that later introduction of CF is associated with a lower BMI or decreased risk of overweight during childhood [12,35]. To the best of our knowledge, CF feeding frequency has not been investigated in childhood, although a previous study reported that CF frequency was not associated with weight gain during infancy [36].

Important strengths of the present study include the prospective data on childhood BMI, as well as information on several infant feeding factors collected during infancy. Moreover, our study population included an ethnic minority group with an increased childhood overweight risk. The fact that mothers were interviewed in their language of choice accounted for differences in language proficiency and established a high response and low dropout rates. Nevertheless, some limitations should be considered. First, the final sample size available for the analyses may be considered small. However, our study includes a sample size that is comparable to or larger than previous studies investigating infant feeding and childhood growth outcomes [14,37,38,39]. Second, due to multiple testing, we also cannot exclude the possibility of spurious findings. Third, although we additionally adjusted for several potential maternal and infant-related confounders, we did not include maternal BMI due to the large number of missing values. However, sensitivity analyses including maternal BMI showed comparable results. Finally, the data regarding the composition of some of the foods consumed were of varying quality, and often not appropriately quantified. Therefore, we lacked information on the intakes of energy and macronutrients. As a result, the CF variety score reflects exposure to a particular food, and not whether a meaningful amount of the food was consumed. Hence, future studies should include detailed information on the composition, as well as the quantities of foods and drinks provided, which may explain the observed associations between the feeding factors and BMI in this study.

To conclude, this study suggests that CF factors, particularly CF variety, explain some of the ethnic differences in BMI between 2 and 5 years of age between children from Dutch and Turkish descent. Therefore, a better understanding of CF practices across ethnic groups is warranted in order to reduce the ethnic inequalities in childhood BMI. Further investigation on the importance of CF variety on childhood BMI is required.

## Figures and Tables

**Table 1:** Characteristics of study population by ethnicity (n = 244). All values are % (n) unless otherwise stated.

	**Dutch (n = 116)**	**Turkish (n = 128)**
	**% (n)**	**% (n)**
**Maternal characteristics**		
Age, years (mean, SD)	32.5 (4.4)	28.7 (4.7)
Parity, primipara	58.6 (68)	28.1 (36)
Smoking during pregnancy	9.5 (11)	21.1 (26)
BMI		
Normal weight	64.3 (54)	28.7 (25)
Overweight (including obesity)	35.7 (30)	71.3 (62)
Unknown	(32)	(41)
Educational level		
Low	4.3 (5)	50.0 (64)
Medium	19.8 (23)	37.5 (48)
High	75.9 (88)	12.5 (16)
**Infant characteristics**		
Birth weight, grams (mean, SD)	3547 (473)	3537 (527)
Gestational age, days (mean, SD)	281 (8.9)	279 (8.1)
Sex, boy	48.3 (56)	52.3 (67)
**Infant feeding factors**		
Full BF duration, months		
<6 months	85.3 (99)	65.6 (84)
≥6 months	14.7 (17)	34.4 (44)
Milk feeding frequency per day at 6 months (mean, SD)	5.0 (1.4)	5.9 (2.2)
CF frequency per day at 6 months (mean, SD)	1.6 (1.0)	2.1 (1.1)
		
Timing of CF introduction		
<6 months	72.4 (84)	64.8 (83)
≥6 months	27.6 (32)	35.2 (45)
	**Dutch (n = 116)**	**Turkish (n = 128)**
	**% (n)**	**% (n)**
CF variety score at 6 months		
1	55.2 (64)	28.6 (38)
2	38.8 (45)	36.7 (47)
≥3	6.0 (7)	35.9 (46)
Types of complementary foods at 6 months ^a^		
Fruits and vegetables	91.8 (123)	85.7 (114)
Dairy products	6.7 (9)	44.4 (59)
Bread, cereals	26.9 (36)	29.3 (39)
Cookies	12.7 (17)	19.5 (26)
Other savory foods	6.5 (6)	39.8 (53)
**Childhood BMI and overweight**		
2 years (n = 250)		
BMI-sds, mean (SD)	0.53 (0.82)	0.56 (1.03)
Overweight ^b^	5.3 (5)	6.2 (7)
3 years (n = 248)		
BMI-sds, mean (SD)	0.18 (0.80)	0.53 (1.01)
Overweight ^b^	4.3 (4)	11.5 (13)
5 years (n = 206)		
BMI-sds, mean (SD)	−0.04 (0.83)	0.63 (1.04)
Overweight ^c^	7.5 (7)	23.5 (25)

BF = breastfeeding; CF = complementary feeding. Missing data (n): maternal BMI (73), smoking (5), maternal age (9), gestational age (4). ^a^ Multiple types of complementary foods can be reported. ^b^ Defined as BMI > 2+ SD above the age- and sex-specific WHO 2006 Child Growth Standards median [24]. ^c^ Defined as BMI > +1 SD above the age- and sex-specific WHO 2007 Child Growth Standards median [23].

**Table 2:** Mean differences in estimated BMI-sds at age 2 years by ethnicity and feeding factors, derived from the LMR (models 1–4) and contribution of infant feeding factors.

	Model 1 ^a^ (Crude Model)	Model 2 ^b^ (Milk Feeding Factors)	Model 3 ^c^ (CF Factors)	Model 4 ^d^ (Model 2 + 3)
	Mean difference (95% CI)			
Ethnicity (Turkish vs. Dutch)	0.08 (−0.16, 0.31)	0.03 (−0.21, 0.27)	0.00 (−0.25, 0.25)	−0.08 (−0.35, 0.18)
Full BF duration (<6 months vs. ≥6 months)		−0.20 (−0.48, 0.08)		−0.18 (−0.46, 0.10)
Milk feeding frequency		0.01 (−0.06, 0.07)		0.02 (−0.05, 0.08)
Timing of CF (<6 months vs. ≥6 months)			−0.03 (−0.26, 0.21)	−0.01 (−0.23, 0.25)
CF variety (2 vs. 1) (≥3 vs. 1)			0.04 (−0.23, −0.32) 0.37 (0.01, 0.72) *	0.06 (−0.22, 0.33) 0.39 (0.03, 0.75) *
CF frequency			−0.08 (−0.19, 0.03)	0.06 (−0.18, 0.05)

BF = breastfeeding; CF = complementary feeding. Data are least-square means and 95% CI. Linear mixed models were used to model mean differences in estimated BMI-sds at age 2 years by ethnicity and the feeding patterns. * *p* < 0.05. ^a^ Model 1: crude model including variables age and ethnicity. ^b^ Model 2: as model 1 and additionally adjusted for the milk feeding factors: full BF duration and milk feeding frequency. ^c^ Model 3: as model 1 and additionally adjusted for the CF factors; timing of CF, CF variety score, CF frequency. ^d^ Model 4: full model, adjusted for all feeding variables (models 1 + 2 + 3).

**Table 3:** Mean differences in estimated BMI-sds at age 3 years by ethnicity and feeding factors, derived from the LMR (models 1–4) and contribution of infant feeding factors.

	Model 1 ^a^ (Crude Model)	Model 2 ^b^ (Milk Feeding Factors)	Model 3 ^c^ (CF Factors)	Model 4 ^d^ (Model 2 + 3)
	Mean difference (95% CI)			
Ethnicity (Turkish vs. Dutch)	0.26 (0.04, 0.48) *	0.22 (−0.01, 0.44)	0.16 (−0.07, 0.40)	0.10 (−0.15, 0.35)
Full BF duration (<6 months vs. ≥6 months)		−0.20 (−0.48, 0.08)		−0.18 (−0.46, 0.10)
Milk feeding frequency		0.01 (−0.06, 0.07)		0.02 (−0.05, 0.08)
Timing of CF (<6 months vs. ≥6 months)			−0.03 (−0.26, 0.21)	−0.01 (−0.23, 0.25)
CF variety score (2 vs. 1) (≥3 vs. 1)			0.03 (−0.23, 0.29) 0.42 (0.08, 0.76) *	0.05 (−0.22, 0.31) 0.44 (0.10, 0.78) *
CF frequency			−0.08 (−0.19, 0.03)	−0.06 (−0.18, 0.05)

BF = breastfeeding; CF = complementary feeding. Data are least-square means and 95% CI. Linear mixed models were used to model mean differences in estimated BMI-sds at age 2 years by ethnicity and the feeding patterns. * *p* < 0.05. ^a^ Model 1: crude model including variables age and ethnicity. ^b^ Model 2: as model 1 and additionally adjusted for the milk feeding factors: full BF duration and milk feeding frequency. ^c^ Model 3: as model 1 and additionally adjusted for the CF factors; timing of CF, CF variety score, CF frequency. ^d^ Model 4: full model, adjusted for all feeding variables (models 1+ 2 + 3).

**Table 4:** Mean differences in estimated BMI-sds at age 5 years by ethnicity and feeding factors, derived from the LMR (models 1–4) and contribution of infant feeding factors.

	Model 1 ^a^ (Crude Model)	Model 2 ^b^ (Milk Feeding Factors)	Model 3 ^c^ (CF Factors)	Model 4 ^d^ (Model 2+3)
	Mean difference (95% CI)			
Ethnicity (Turkish vs. Dutch)	0.63 (0.39, 0.88) ***	0.59 (0.33, 0.84) ***	0.50 (0.24, 0.77) ***	0.47 (0.20, 0.75) **
Full BF duration (<6 months vs. ≥6 months)		−0.20 (−0.48, 0.08)		−0.18 (−0.46, 0.10)
Milk feeding frequency		0.01 (−0.06, 0.07)		0.02 (−0.05, 0.08)
Timing of CF (<6 months vs. ≥6 months)			−0.03 (−0.26, 0.21)	−0.01 (−0.23, 0.25)
CF variety score (2 vs. 1) (≥3 vs. 1)			(−0.28, 0.30) 0.53 (0.15, 0.90) **	0.02 (−0.27, 0.31) 0.55 (0.17, 0.92) **
CF frequency			−0.08 (−0.19, 0.03)	−0.06 (−0.18, 0.05)

BF = breastfeeding; CF = complementary feeding. Data are least-square means and 95% CI. Linear mixed models were used to model mean differences in estimated BMI-sds at age 2 years by ethnicity and the feeding patterns. ** *p* < 0.01. *** *p* < 0.001. ^a^ Model 1: crude model including variables age and ethnicity. ^b^ Model 2: as model 1 and additionally adjusted for the milk feeding factors: full BF duration and milk feeding frequency. ^c^ Model 3: as model 1 and additionally adjusted for the CF factors; timing of CF, CF variety score, CF frequency. ^d^ Model 4: full model, adjusted for all feeding variables (models 1+ 2 + 3).

## Data Availability

The data are not publicly available due to ethical reasons.

## Supplementary Materials

The following are available online at https://www.mdpi.com/article/10.3390/nu13072291/s1, Figure S1: Flowchart of the study participants; Table S1: Mean differences in estimated BMI-sds at ages 2, 3 and 5 by ethnicity and feeding factors (n = 229), derived from the LMR model 5; Table S2: Mean differences in estimated BMI-sds at ages 2, 3 and 5 years by ethnicity and feeding factors (n = 156), derived from the LMR (model 6).

## References

[1] Di Cesare M., Sorić M., Bovet P., Miranda J.J., Bhutta Z., Stevens G.A., Laxmaiah A., Kengne A.P., Bentham J. (2019). The epidemiological burden of obesity in childhood: A worldwide epidemic requiring urgent action. BMC Med..

[2] de Wilde J.A., Meeuwsen R.C., Middelkoop B.J. (2018). Growing ethnic disparities in prevalence of overweight and obesity in children 2–15 years in the Netherlands. Eur. J. Public Health.

[3] van Dommelen P., Schonbeck Y., HiraSing R.A., van Buuren S. (2015). Call for early prevention: Prevalence rates of overweight among Turkish and Moroccan children in The Netherlands. Eur. J. Public Health.

[4] Statistics Netherlands Lengte en Gewicht van Personen, Ondergewicht en Overgewicht; Vanaf 1981. https://opendata.cbs.nl/statline/#/CBS/nl/dataset/81565NED/table?fromstatweb.

[5] Hof M.H., van Dijk A.E., van Eijsden M., Vrijkotte T.G., Zwinderman A.H. (2011). Comparison of growth between native and immigrant infants between 0-3 years from the Dutch ABCD cohort. Ann. Hum. Biol..

[6] Koletzko B., von Kries R., Closa R., Escribano J., Scaglioni S., Giovannini M., Beyer J., Demmelmair H., Anton B., Gruszfeld D. (2009). Can infant feeding choices modulate later obesity risk?. Am. J. Clin. Nutr..

[7] Rzehak P., Oddy W.H., Mearin M.L., Grote V., Mori T.A., Szajewska H., Shamir R., Koletzko S., Weber M., Beilin L.J. (2017). Infant feeding and growth trajectory patterns in childhood and body composition in young adulthood. Am. J. Clin. Nutr..

[8] D’Auria E., Borsani B., Pendezza E., Bosetti A., Paradiso L., Zuccotti G.V., Verduci E. (2020). Complementary Feeding: Pitfalls for Health Outcomes. Int. J. Environ. Res. Public Health.

[9] English L.K., Obbagy J.E., Wong Y.P., Butte N.F., Dewey K.G., Fox M.K., Greer F.R., Krebs N.F., Scanlon K.S., Stoody E.E. (2019). Timing of introduction of complementary foods and beverages and growth, size, and body composition: A systematic review. Am. J. Clin. Nutr..

[10] Patro-Gołąb B., Zalewski B.M., Polaczek A., Szajewska H. (2019). Duration of Breastfeeding and Early Growth: A Systematic Review of Current Evidence. Breastfeed. Med..

[11] Horta B.L., Loret de Mola C., Victora C.G. (2015). Long-term consequences of breastfeeding on cholesterol, obesity, systolic blood pressure and type 2 diabetes: A systematic review and meta-analysis. Acta Paediatr..

[12] Seach K.A., Dharmage S.C., Lowe A.J., Dixon J.B. (2010). Delayed introduction of solid feeding reduces child overweight and obesity at 10 years. Int. J. Obes..

[13] Vail B., Prentice P., Dunger D.B., Hughes I.A., Acerini C.L., Ong K.K. (2015). Age at Weaning and Infant Growth: Primary Analysis and Systematic Review. J. Pediatr..

[14] Gunnarsdottir I., Schack-Nielsen L., Michaelsen K.F., Sørensen T.I., Thorsdottir I. (2010). Infant weight gain, duration of exclusive breast-feeding and childhood BMI—Two similar follow-up cohorts. Public Health Nutr..

[15] Fernandez C., Kasper N.M., Miller A.L., Lumeng J.C., Peterson K.E. (2016). Association of Dietary Variety and Diversity With Body Mass Index in US Preschool Children. Pediatrics.

[16] Gridneva Z., Rea A., Hepworth A.R., Ward L.C., Lai C.T., Hartmann P.E., Geddes D.T. (2018). Relationships between Breastfeeding Patterns and Maternal and Infant Body Composition over the First 12 Months of Lactation. Nutrients.

[17] Santorelli G., Fairley L., Petherick E.S., Cabieses B., Sahota P. (2014). Ethnic differences in infant feeding practices and their relationship with BMI at 3 years of age—Results from the Born in Bradford birth cohort study. Br. J. Nutr..

[18] de Hoog M.L., van Eijsden M., Stronks K., Gemke R.J., Vrijkotte T.G. (2011). The role of infant feeding practices in the explanation for ethnic differences in infant growth: The Amsterdam Born Children and their Development study. Br. J. Nutr..

[19] Sirkka O., Vrijkotte T., Halberstadt J., Abrahamse-Berkeveld M., Hoekstra T., Seidell J., Olthof M. (2018). Prospective associations of age at complementary feeding and exclusive breastfeeding duration with body mass index at 5-6 years within different risk groups. Pediatr. Obes..

[20] van Eijsden M., Meijers C.M., Jansen J.E., de Kroon M.L., Vrijkotte T.G. (2015). Cultural variation in early feeding pattern and maternal perceptions of infant growth. Br. J. Nutr..

[21] Stronks K., Kulu-Glasgow I., Agyemang C. (2009). The utility of ‘country of birth’ for the classification of ethnic groups in health research: The Dutch experience. Ethn. Health.

[22] WHO (2002). Complementary Feeding: Report of the Global Consultation and Summary of Guiding Principles for Complementary Feeding of the Breastfed Child.

[23] WHO Multicentre Growth Reference Study Group (2006). WHO Child Growth Standards based on length/height, weight and age. Acta Paediatr. Suppl..

[24] de Onis M., Onyango A.W., Borghi E., Siyam A., Nishida C., Siekmann J. (2007). Development of a WHO growth reference for school-aged children and adolescents. Bull. World Health Organ..

[25] Papageorghiou A.T., Ohuma E.O., Altman D.G., Todros T., Cheikh Ismail L., Lambert A., Jaffer Y.A., Bertino E., Gravett M.G., Purwar M. (2014). International standards for fetal growth based on serial ultrasound measurements: The Fetal Growth Longitudinal Study of the INTERGROWTH-21st Project. Lancet.

[26] de Hoog M.L., van Eijsden M., Stronks K., Gemke R.J., Vrijkotte T.G. (2011). Overweight at age two years in a multi-ethnic cohort (ABCD study): The role of prenatal factors, birth outcomes and postnatal factors. BMC Public Health.

[27] van Rossem L., Hafkamp-de Groen E., Jaddoe V.W., Hofman A., Mackenbach J.P., Raat H. (2014). The role of early life factors in the development of ethnic differences in growth and overweight in preschool children: A prospective birth cohort. BMC Public Health.

[28] Schack-Nielsen L., Sorensen T., Mortensen E.L., Michaelsen K.F. (2010). Late introduction of complementary feeding, rather than duration of breastfeeding, may protect against adult overweight. Am. J. Clin. Nutr..

[29] Nicklaus S. (2009). Development of food variety in children. Appetite.

[30] Vadiveloo M., Dixon L.B., Parekh N. (2013). Associations between dietary variety and measures of body adiposity: A systematic review of epidemiological studies. Br. J. Nutr..

[31] Yan J., Liu L., Zhu Y., Huang G., Wang P.P. (2014). The association between breastfeeding and childhood obesity: A meta-analysis. BMC Public Health.

[32] Besharat Pour M., Bergstrom A., Bottai M., Magnusson J., Kull I., Moradi T. (2017). Age at adiposity rebound and body mass index trajectory from early childhood to adolescence; differences by breastfeeding and maternal immigration background. Pediatr. Obes..

[33] Fairley L., Santorelli G., Lawlor D.A., Bryant M., Bhopal R., Petherick E.S., Sahota P., Greenwood D.C., Hill A.J., Cameron N. (2015). The relationship between early life modifiable risk factors for childhood obesity, ethnicity and body mass index at age 3 years: Findings from the Born in Bradford birth cohort study. BMC Obes..

[34] Pearce J., Taylor M.A., Langley-Evans S.C. (2013). Timing of the introduction of complementary feeding and risk of childhood obesity: A systematic review. Int. J. Obes..

[35] Sun C., Foskey R.J., Allen K.J., Dharmage S.C., Koplin J.J., Ponsonby A.L., Lowe A.J., Matheson M.C., Tang M.L., Gurrin L. (2016). The Impact of Timing of Introduction of Solids on Infant Body Mass Index. J. Pediatr..

[36] WHO Working Group on the Growth Reference Protocol WHO Task Force on Methods for the Natural Regulation of Fertility (2002). Growth of healthy infants and the timing, type, and frequency of complementary foods. Am. J. Clin. Nutr..

[37] Imai C.M., Gunnarsdottir I., Thorisdottir B., Halldorsson T.I., Thorsdottir I. (2014). Associations between infant feeding practice prior to six months and body mass index at six years of age. Nutrients.

[38] Butte N.F., Wong W.W., Hopkinson J.M., Smith E.O., Ellis K.J. (2000). Infant feeding mode affects early growth and body composition. Pediatrics.

[39] Lourenço B.H., Villamor E., Augusto R.A., Cardoso M.A. (2015). Influence of early life factors on body mass index trajectory during childhood: A population-based longitudinal analysis in the Western Brazilian Amazon. Matern. Child. Nutr..

