# Supplementary material for: Infant Feeding and Ethnic Differences in Body Mass Index during Childhood: A Prospective Study
Source: Nutrients. 2021 Jul 1;13(7):2291. doi: 10.3390/nu13072291 (PMC8308235; doi:10.3390/nu13072291)
Supplement: Supplementary file 1 [file nutrients-13-02291-s001.zip › nutrients-1281029-supplementary.pdf]

Supplementary material

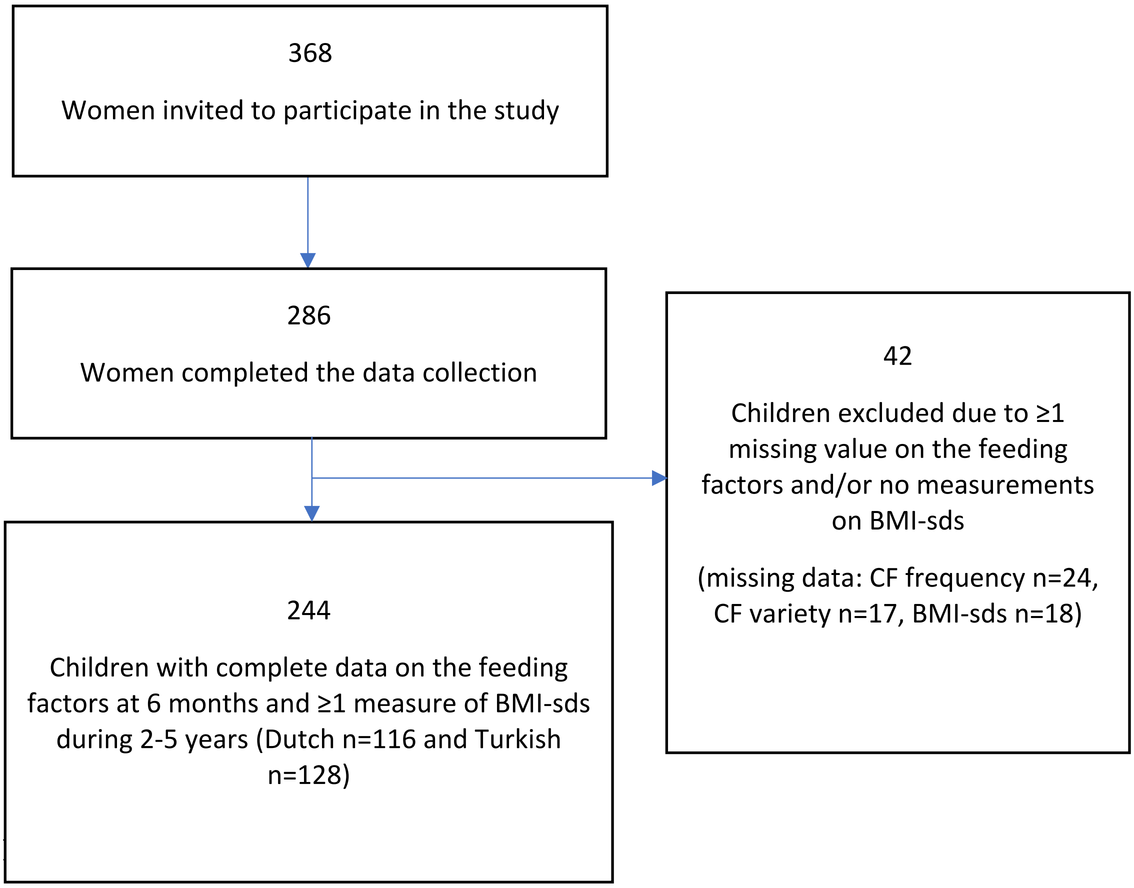

Figure S1. Flowchart of the study participants.

Table S1. Mean differences in estimated BMI-sds at ages 2, 3 and 5 by ethnicity and feeding factors (n=229), derived from the LMR model 5.

	Model 5 [a]		
	**2 years**	**3 years**	**5 years**
	**Mean difference (95% CI)**		
Ethnicity (Turkish vs. Dutch)	-0.13 (-0.42, 0.17)	0.03 (-0.26, 0.31)	0.33 (0.02, 0.64)*
Full BF duration (<6 months vs. ≥6 months)	-0.11 (-0.40, 0.17)	-0.11 (-0.40, 0.17)	-0.11 (-0.40, 0.17)
Milk feeding frequency	0.03 (-0.03, 0.10)	0.03 (-0.03, 0.10)	0.03 (-0.03, 0.10)
Timing of CF (<6 months vs. ≥6 months)	-0.05 (-0.29, 0.19)	-0.05 (-0.29, 0.19)	-0.05 (-0.29, 0.19)
CF variety score			
(2 vs. 1)	0.11 (-0.16, 0.38)	0.11 (-0.15, 0.37)	0.12 (-0.17, 0.41)
(≥3 vs. 1)	0.37 (0.01, 0.73)*	0.41 (0.07, 0.76)*	0.49 (0.12, 0.87)*
CF frequency	-0.05 (-0.16, 0.06)	-0.05 (-0.16, 0.06)	-0.05 (-0.16, 0.06)

BF = breastfeeding; CF = complementary feeding. Data are least-square means and 95% CI. Linear mixed models were used to model mean differences in estimated BMI-sds at age 2, 3 and 5 years by ethnicity and the feeding patterns. * P<0.05. [a] Adjusted for all infant feeding factors and the covariates (maternal: age, smoking, parity; child: sex, gestational age, birth weight-sds).

Table S2. Mean differences in estimated BMI-sds at ages 2, 3 and 5 years by ethnicity and feeding factors (n=156), derived from the LMR (model 6).

	**Model 6 [a]**		
	**2 years**	**3 years**<br>**Mean difference (95% CI)**	**5 years**
Ethnicity<br>(Turkish vs. Dutch)	-0.24 (-0.60, 0.12)	0.08 (-0.26, 0.43)	0.23 (-0.14, 0.60)
Full breastfeeding duration<br>(<6 months vs. ≥6 months)	-0.22 (-0.58, 0.14)	-0.22 (-0.58, 0.14)	-0.22 (-0.58, 0.14)
Milk feeding frequency	0.02 (-0.06, 0.10)	0.02 (-0.06, 0.10)	0.02 (-0.06, 0.10)
Timing of complementary feeding<br>(<6 months vs. ≥6 months)	-0.05 (-0.33, 0.22)	-0.05 (-0.33, 0.22)	-0.05 (-0.33, 0.22)
Complementary feeding variety score			
(2 vs. 1)	0.05 (-0.26, 0.37)	0.08 (-0.22, 0.38)	0.14 (-0.19, 0.48)
(≥3 vs. 1)	0.35 (-0.11, 0.81)	0.39 (-0.05, 0.83)	0.48 (0.01, 0.96)*
CF frequency	-0.02 (-0.16, 0.12)	-0.02 (-0.16, 0.12)	-0.02 (-0.16, 0.12)

BF = breastfeeding; CF = complementary feeding. Data are least-square means and 95% CI. Linear mixed models were used to model mean differences in estimated BMI-sds at age 2, 3 and 5 years by ethnicity and the feeding patterns. * P<0.05. [a] Model includes model 5 (all infant feeding factors and maternal: age, smoking, parity; child: gestational age, birth weight) and maternal BMI.
